# HMG20B stabilizes association of LSD1 with GFI1 on chromatin to confer transcription repression and leukemia cell differentiation block

**DOI:** 10.1038/s41388-022-02471-y

**Published:** 2022-09-28

**Authors:** Alba Maiques-Diaz, Luciano Nicosia, Naseer J. Basma, Isabel Romero-Camarero, Francesco Camera, Gary J. Spencer, Fabio M. R. Amaral, Fabrizio Simeoni, Bettina Wingelhofer, Andrew J. K. Williamson, Andrew Pierce, Anthony D. Whetton, Tim C. P. Somervaille

**Affiliations:** 1 Leukaemia Biology Laboratory, Cancer Research UK Manchester Institute, The University of Manchester, Manchester Cancer Research Centre Building, 555 Wilmslow Road, Manchester, M20 4GJ UK; 2 Stem Cell and Leukaemia Proteomics Laboratory, Manchester Academic Health Science Centre, The University of Manchester, Wolfson Molecular Imaging Centre, 27 Palatine Road, Manchester, M20 3LJ UK; 3 School of Medical and Health Sciences, College of Human Sciences, Fron Heulog Bangor University, Bangor, LL57 2TH UK; 4 School of Veterinary Medicine and School of Biosciences and Medicine, University of Surrey, VSM Building, University of Surrey, Guildford, GU2 7AL UK

**Keywords:** Acute myeloid leukaemia, Proteomics, Mechanisms of disease

## Abstract

Pharmacologic inhibition of LSD1 induces molecular and morphologic differentiation of blast cells in acute myeloid leukemia (AML) patients harboring *MLL* gene translocations. In addition to its demethylase activity, LSD1 has a critical scaffolding function at genomic sites occupied by the SNAG domain transcription repressor GFI1. Importantly, inhibitors block both enzymatic and scaffolding activities, in the latter case by disrupting the protein:protein interaction of GFI1 with LSD1. To explore the wider consequences of LSD1 inhibition on the LSD1 protein complex we applied mass spectrometry technologies. We discovered that the interaction of the HMG-box protein HMG20B with LSD1 was also disrupted by LSD1 inhibition. Downstream investigations revealed that HMG20B is co-located on chromatin with GFI1 and LSD1 genome-wide; the strongest HMG20B binding co-locates with the strongest GFI1 and LSD1 binding. Functional assays demonstrated that HMG20B depletion induces leukemia cell differentiation and further revealed that HMG20B is required for the transcription repressor activity of GFI1 through stabilizing LSD1 on chromatin at GFI1 binding sites. Interaction of HMG20B with LSD1 is through its coiled-coil domain. Thus, HMG20B is a critical component of the GFI1:LSD1 transcription repressor complex which contributes to leukemia cell differentiation block.

## Introduction

Epigenetic and transcription factor complexes are emerging therapeutic targets across a range of human malignancies. The histone demethylase LSD1 (Lysine Specific Demethylase 1, also known as KDM1A) has been the focus of recent interest in view of its high-level expression in acute myeloid leukemia (AML) and a range of solid tumors, as well as encouraging pre-clinical and early phase clinical studies using candidate inhibitors [1–4]. LSD1 forms a corepressor complex with RCOR1 (CoREST), histone deacetylase (HDAC1/2), and other components [5] and has enzymatic capacity to demethylate monomethyl and dimethyl lysine 4 of histone H3 (H3K4) in a flavin adenine dinucleotide (FAD) dependent manner [6]. In addition, LSD1 has a critical scaffolding function. It binds the N-terminal sequence of SNAG domain transcription factors such as GFI1 and SNAIL through a peptide binding cleft formed by the amine oxidase domain [7]; this interaction is essential for the transcription repressor function of these DNA-binding proteins. SNAG domain sequences are structural mimics of the N-terminal tail of Histone H3 [7] and when LSD1’s binding cleft is occupied there is no access for the histone tail to the catalytic activity of LSD1. In leukemia cells, recruitment of LSD1 to chromatin genome-wide is overwhelmingly at sites of GFI1 binding [8] and release of LSD1 from GFI1-occupied sites by disruption of protein:protein interaction is required for LSD1 inhibitor-induced leukemia cell differentiation [8]. Pharmacologic inhibition of LSD1 has also proven to be effective in pre-clinical models of SNAG transcription factor-driven neoplasia [8–14].

GFI1 is important in normal and leukemic hematopoiesis where it is required for the functional integrity of hematopoietic stem cells, lymphoid development, and generation of neutrophils [15–17]. Mutations in *GFI1* cause severe congenital neutropenia [18] and *GFI1* expression levels have been variably linked to prognosis in AML according to disease subtype [19]. The interaction between GFI1 and LSD1 can be disrupted by a single SNAG domain point mutation (P2A) which inactivates GFI1’s function as a transcriptional repressor [20]. Despite important prior insights, the role of many of the proteins found in the LSD1 complex, and how they interact at sites of GFI1 binding to confer transcription repression, remains to be elucidated [21].

In this study, our goal was to identify LSD1 protein binding partners that facilitate the stability of LSD1’s interaction with GFI1 on chromatin. For this purpose, we used *MLL*-rearranged leukemia cell models which are dependent on the physical interaction of LSD1 with GFI1 to maintain their proliferative, undifferentiated cellular state [4, 8].

## Results

### Pharmacologic inhibition of LSD1 disrupts its interaction with the DNA-binding cofactor HMG20B

**Fig. 1:**
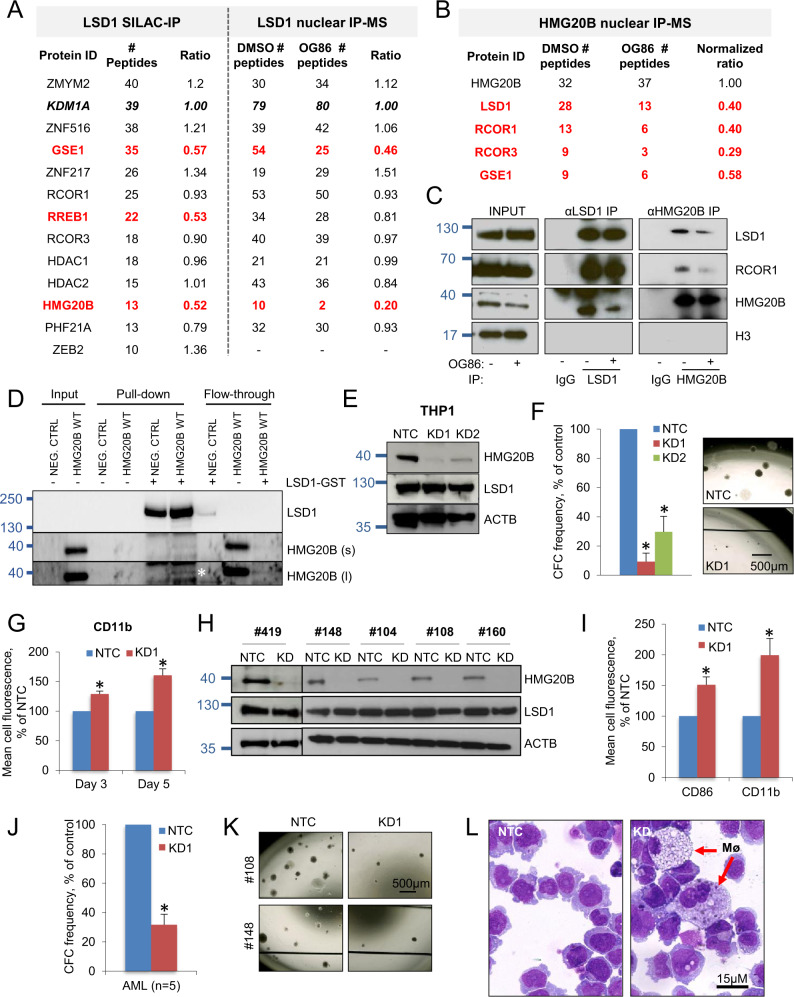
Pharmacological inhibition of LSD1 destabilizes its interaction with HMG20B. **A**–**C** THP1 AML cells were treated with 250 nM OG86 or DMSO vehicle for 48 h. Proteins known to form part of an LSD1-containing complex and identified by mass spectrometry techniques following **A** LSD1 or **B** HMG20B immunoprecipitation (IP) are shown. Proteins in red exhibit reduced interaction in the presence of OG86. **C** IP westerns of nuclear lysates using the indicated antibodies. **D** IP western shows interaction (white asterisk) of in vitro synthesized HMG20B with purified GST-LSD1, and efficient depletion of HMG20B from flow through. s = short exposure; l = long exposure. **E**–**G** THP1 AML cells were infected with lentiviral vectors targeting *HMG20B* for KD, or a non-targeting control (NTC), with puromycin drug resistance as the selectable marker. **E** Western blots show expression of the indicated proteins three days after infection. **F** Mean + SEM colony-forming cell (CFC) frequencies relative to controls after ten days in semisolid culture (*n* = 3); right panels show representative colonies. **G** Mean+SEM CD11b cell fluorescence relative to control as determined by flow cytometry three and five days after initiation of knockdown (*n* = 2). **H**–**L** Five independent primary *MLL*-rearranged AML cells were infected with lentiviral vectors targeting *HMG20B* for KD (KD1 construct used), or a NTC, with puromycin drug resistance as the selectable marker (# refers to Biobank identifier). **H** Western blots show the indicated proteins three days after infection. **I** Mean + SEM CD11b and CD86 cell fluorescence relative to control 5 days after initiation of knockdown (*n* = 5 separate patient samples). **J** Mean + SEM CFC frequencies after ten days in semisolid culture (*n* = 5 separate patient samples). **K** Representative images of colonies. **L** Representative images of cytospin preparations. Mø, macrophage. * indicates *P* < 0.05 by unpaired *t*-test or, in (**F**), by one-way ANOVA with a Tukey post hoc test.

To identify proteins displaced from physical interaction with LSD1 following treatment of AML cells with OG86 (Oryzon Genomics compound #86; trans-N-((2-methoxypyridin-3-yl)methyl)-2-phenylcyclopropan-1-amine) which is a representative tranylcypromine-derivative inhibitor of LSD1, we used both Stable Isotope Labeling by Amino acids in cell Culture (SILAC) and Immunoprecipitation-Mass Spectrometry (IP-MS) as relative quantitation proteomics approaches (Fig. 1A) [2, 8]. THP1 AML cells were selected because they exhibit a t(9;11) *MLL* gene rearrangement and respond to LSD1 inhibition in a similar manner to primary patient *MLL*-translocated AML cells, with differentiation and loss of clonogenic activity [4, 8]. For SILAC, cells were grown in heavy or light media for seven passages, treated with OG86 250 nM or DMSO vehicle respectively for 48 h and then protein lysates from both conditions were combined and subjected to endogenous LSD1 immunoprecipitation (Fig. 1A; Table S1). In parallel, we performed LSD1 MS-IP using nuclear-enriched protein lysates from THP1 AML cells exposed to either OG86 250 nM or DMSO, also for 48 h (Fig. 1A; Table S2). Both IP experiments identified proteins previously reported in multiple studies to be found in complex with LSD1 [1]. In keeping with our prior findings [8], we found no evidence that OG86 reduced the interaction of LSD1 with complex components RCOR1/2/3 and HDAC1/2. Likewise, the same was the case for PHF21A and zinc finger proteins ZMYM2/3, ZNF217, ZNF516, and ZEB2. However, in both experiments, we observed reduced interaction with LSD1 of the high-mobility group protein HMG20B (also known as BRAF35) which is known to interact with LSD1 and with DNA in a non-sequence-specific manner [22, 23], as well as the coiled-coil domain protein GSE1. The ratio of RREB1 binding was reduced in the SILAC experiment but to a lesser extent in the MS-IP experiment. We focused our attention on HMG20B, given its role in repressing erythroid differentiation [24] and the reported roles of its paralog HMG20A in SNAG domain transcription factor repressive complexes associated with SNAI1 [25], INSM1 [26], and GFI1B [27] in different cellular contexts.

To confirm the reduced interaction of HMG20B with LSD1 following LSD1 inhibition, we performed HMG20B MS-IP using nuclear-enriched protein lysates from THP1 AML cells exposed to either OG86 250 nM or DMSO for 48 h (Fig. 1B and Table S3) and reciprocal IP-western analysis for both HMG20B and LSD1 (Fig. 1C). HMG20B IP-MS confirmed an interaction of HMG20B with LSD1, RCOR1/3 and GSE1 (Fig. 1B and Table S3) although not zinc finger proteins ZMYM2/3, ZNF217, ZNF516, and ZEB2. Both MS and IP-western approaches confirmed that LSD1 inhibition reduced the physical association of HMG20B with LSD1 and RCOR1. We confirmed that the interaction of HMG20B with LSD1 was direct. In vitro transcribed and translated HMG20B interacted with, and was efficiently depleted from solution by, purified GST-LSD1 (Fig. 1D). Thus, we concluded that an exemplar tranylcypromine-derivative inhibitor of LSD1 destabilizes the physical association of LSD1/RCOR1 with its associated complex component HMG20B.

### Depletion of HMG20B induces leukemia cell differentiation

To explore the functional role of HMG20B, we induced *HMG20B* knockdown (KD) in THP1 AML cells and in five primary patient AML samples with *MLL* gene rearrangements. In THP1 cells *HMG20B* KD resulted in upregulation of the myeloid differentiation marker CD11b and reduced clonogenic activity in semisolid culture (Fig. 1E–G). We made similar observations in primary patient *MLL* leukemia cells with upregulation of differentiation markers CD11b and CD86, reduced clonogenic activity, increased maturation of cells to mature macrophages (Fig. 1H–L; Table S4) and a G1 cell cycle arrest without significant enhancement of apoptosis (Fig. S1A, B). These phenotypic observations were very similar to those we reported following pharmacological inhibition of LSD1 in cell line and patient cells [8], in keeping with the concept that HMG20B has an important role in the LSD1 complex.

### Close association of HMG20B on chromatin with LSD1 and GFI1

**Fig. 2:**
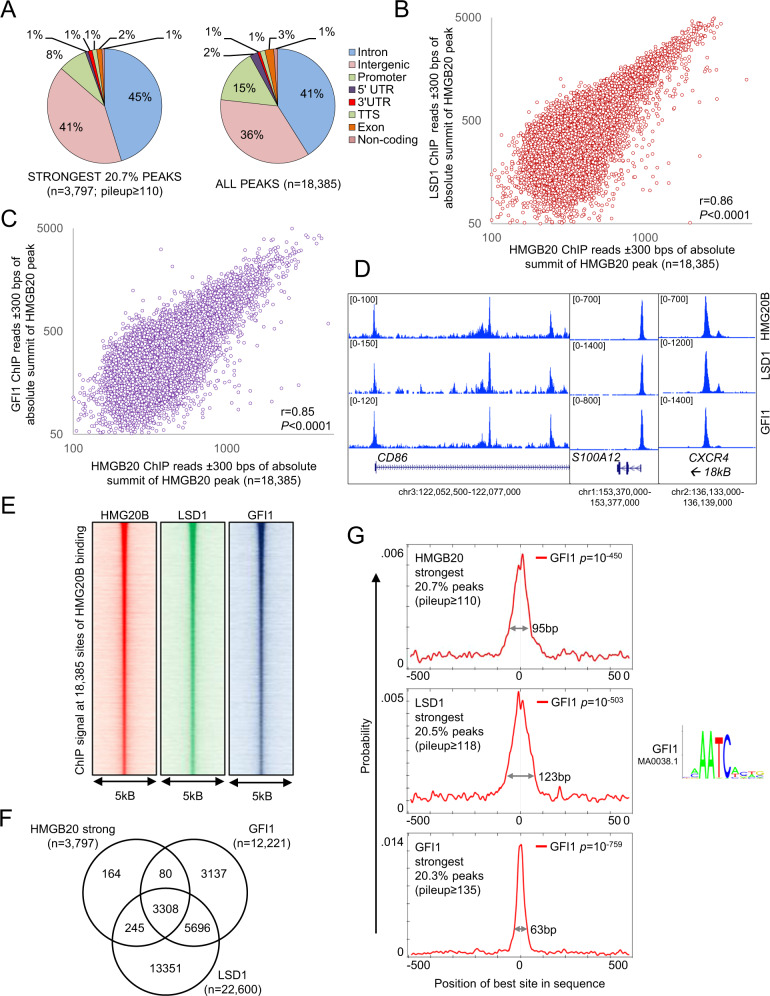
HMG20B cooccupies chromatin with GFI1 and LSD1. **A** Pie charts show genome annotations for the strongest (left) or all (right) HMG20B peaks. UTR untranslated region, TTS transcription termination sequence. Dot plots show correlation between HMG20B ChIP signal and **B** LSD1 ChIP signal or **C** GFI1 ChIP signal. **D** Exemplar ChIPseq tracks. **E** Heatmaps show HMG20B, LSD1 and GFI1 ChIP signal ±2.5 kb surrounding absolute summit of all HMG20B peaks ranked by peak strength. **F** Venn diagram shows intersection of strong HMG20B peaks with LSD1 and GFI1 binding peaks. **G** MEME-ChIP GFI1 motif enrichment plots surrounding absolute summits of strong HMG20B, LSD1 or GFI1 binding peaks.

We previously reported that LSD1 near exclusively co-locates on chromatin with the transcription factor GFI1 in THP1 AML cells, with the strength of LSD1 chromatin immunoprecipitation (ChIP) signal correlating strongly and positively with GFI1 ChIP signal [8]. To evaluate the co-localization of endogenous HMG20B in relation to endogenous LSD1 and GFI1 genome-wide, we performed ChIP followed by next generation sequencing (ChIPseq) for the three proteins. After excluding blacklisted genomic regions prone to artifact and making use of stringent threshold criteria (called peaks had pileup value ≥50 and fold enrichment over input ≥ 5), MACS2 [28] identified 18,385 HMG20B peaks, 22,600 LSD1 peaks and 12,221 GFI1 peaks. As we previously reported for LSD1 and GFI1 [8], whether all peaks were considered, or the strongest 20.7% (i.e. those with pileup values ≥ 110; *n* = 3797), HMG20B binding was distributed predominantly over intronic and intergenic regions versus promoter regions (Fig. 2A), consistent with putative roles at enhancers. Genome-wide, HMG20B peaks were co-located with GFI1 and LSD1 binding peaks, and stronger HMG20B binding correlated positively and significantly with stronger GFI1 and LSD1 binding (Fig. 2B–E). Indeed 91.0% and 94.8% respectively of strong HMG20B peaks overlapped with either a GFI1 or an LSD1 binding peak (Fig. 2F) (i.e. absolute summit of peaks separated by ≤ 100 base pairs). In keeping with the close physical association of HMG20B with both GFI1 and LSD1 on chromatin, MEME-ChIP [29] confirmed that genomic sequences surrounding the absolute summits of the strongest 20.7% of HMG20B binding peaks were strongly enriched for the GFI1 consensus binding motif (Fig. 2G).

Interestingly, there were notable differences in the distribution of GFI1 motifs according to whether the absolute summits of strong GFI1 peaks, strong LSD1 peaks or strong HMG20B peaks were considered (Fig. 2G). Relative to the absolute summit of strong GFI1 peaks (i.e., the strongest 20.3%, with pileup values ≥ 135; *n* = 2479), the enriched region of GFI1 transcription factor binding motifs was narrow (63 base pairs), with the apex of the motif probability graph coinciding with the absolute summit of GFI1 ChIPseq peaks (Fig. 2G, bottom panel). By contrast, relative to the absolute summit of strong HMG20B or strong LSD1 ChIPseq peaks (i.e. the strongest 20.5%, with pileup values ≥118; *n* = 4641), the enriched region of GFI1 transcription factor binding motifs was broader (95 or 123 base pairs respectively) with, in addition, the apex of the GFI1 motif probability curve offset from the absolute summit of HMG20B or LSD1 ChIPseq peaks by a modal value of 8–10 base pairs on each side (Fig. 2G, upper and middle panels). These motif distribution plots are consistent with the GFI1/LSD1/HMG20B repressor complex being recruited to chromatin through sequence-specific binding of GFI1, with HMG20B serving an accessory, stabilizing function through binding of its HMG-box to nearby bent, kinked or unwound non-B-type DNA structures [30].

**Fig. 3:**
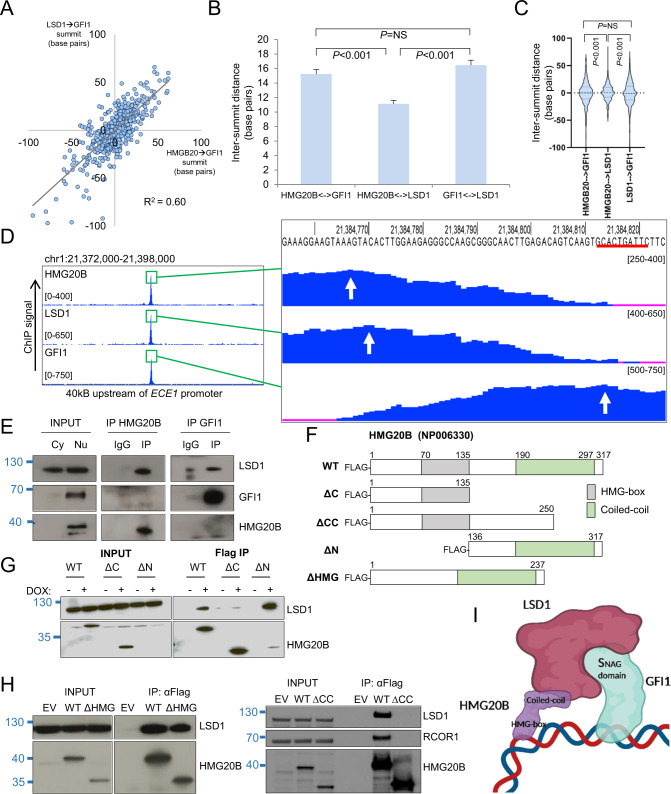
Co-localization pattern of HMG20B, LSD1 and GFI1 on chromatin. **A** Dot plot show base pair distances between the absolute summits of HMG20B and GFI1 binding peaks (with pileup value ≥300) versus base pair distances between the absolute summits of LSD1 and GFI1 binding peaks. **B** Bar graph shows mean+SEM intersummit base pair distances between the indicated factor binding peaks. **C** Violin plots show distribution, median (thick dotted line), and interquartile range (light dotted lines) for intersummit base pair distances between the indicated factor binding peaks. **D** Exemplar ChIPseq tracks. Red line indicates GFI1 binding motif. White arrows indicate called peak summits. **E** Western shows nuclear immunoprecipitation for the indicated proteins in THP1 AML cells. Cy cytoplasmic, Nu nuclear. **F** HMG20B constructs. WT wild type. **G**, **H** Westerns show doxycycline-induced (DOX) expression of HMG20B constructs and αFlag immunoprecipitations in THP1 AML cells. **I** Proposed model of chromatin binding (created with BioRender). For **B** and **C**, one-way ANOVA with Tukey post hoc test was used to determine statistical significance.

Next we determined base pair distances between the summits of strong GFI1 peaks and co-located strong HMG20B and strong LSD1 peaks. To maximize the signal:noise ratio we focused on the strongest 526 GFI1 peaks (i.e. those with pileup values ≥ 300) where there were also overlapping strong HMG20B and LSD1 peaks, all with absolute summits within 100 base pairs of one other. The base pair distances from HMG20B peak summits to GFI1 peak summits were strongly correlated with those from LSD1 peak summits to GFI1 peak summits (Fig. 3A). In contrast there was little correlation between HMG20B→LSD1 versus HMG20B→GFI1 base pair distances (Fig. S2A), nor HMG20B→LSD1 versus LSD1→GFI1 base pair distances (Fig. S2B). The mean base pair distance between summits of HMG20B and LSD1 peaks was significantly lower than that between either HMG20B and GFI1 peaks, or LSD1 and GFI1 peaks (Fig. 3B). There was no significant difference in base pair distances between summits of HMG20B and GFI1 peaks versus distances between LSD1 and GFI1 peaks (Fig. 3B–D). These data suggest that HMG20B and LSD1 co-occupy chromatin to one side or the other of GFI1.

### HMG20B interacts with LSD1 through its coiled-coil domain

To further evaluate the interaction between HMG20B, LSD1, and GFI1, we performed nuclear immunoprecipitation experiments in the presence of benzonase to digest nucleic acid. While immunoprecipitation of both HMG20B and GFI1 pulled down LSD1, IP of HMG20B did not pulldown GFI1 and vice versa (Fig. 3E), suggesting that stable association of HMG20B with GFI1 on chromatin requires intervening DNA. To determine which domain of HMG20B recruits LSD1 to chromatin we generated and expressed HMG20B deletion mutants (Fig. 3F). While full-length HMG20B pulled down LSD1, deletion mutants lacking the coiled-coil domain (ΔC or ΔCC) did not (Fig. 3G, H). In contrast, mutants lacking the DNA-binding HMG-box readily interacted with LSD1 (Fig. 3G, H), despite the low-level expression of the full N-terminal deletion mutant following doxycycline induction, likely due to reduced protein stability (Fig. 3G). HMG20B and GFI1 therefore bind DNA close to one another on chromatin through, respectively, the HMG box of HMG20B and the zinc finger domain of GFI1. LSD1 interacts through two points of contact: the coiled-coil domain of HMG20B and the N-terminal SNAG domain of GFI1 [7] (Fig. 3I).

### Functional recruitment of LSD1 to chromatin by a GFI1 DNA-binding domain:HMG20B fusion protein

**Fig. 4:**
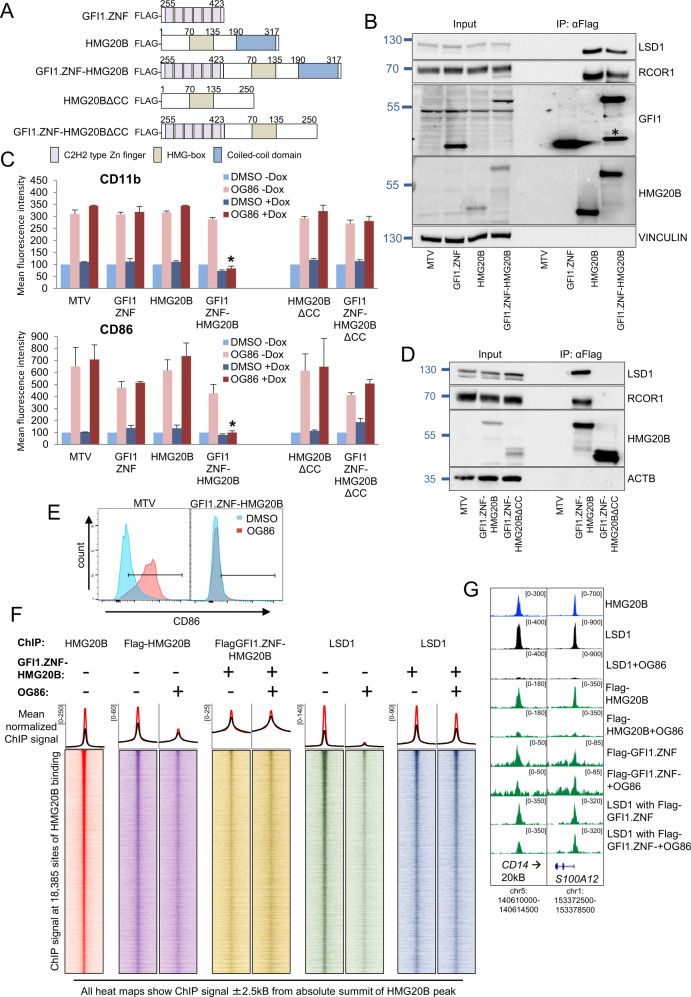
Functional recruitment of LSD1 to chromatin by a GFI1 DNA-binding domain:HMG20B fusion protein. **A** Construct maps. **B** Western shows doxycycline-induced expression of the indicated constructs and αFlag IPs in THP1 AML cells. * Indicates degradation product. **C** THP1 AML cells were infected with lentiviruses expressing the indicated doxycycline-inducible (Dox) GFI1 fusion or control constructs and then treated with 250 nM OG86 or DMSO vehicle. Bar graphs indicate means + SEM fluorescence intensity for the indicated markers, as determined by flow cytometry, in the indicated conditions 24 h later (*n* = 3). * indicates *P* < 0.05 for the indicated condition versus all other OG86 + Dox conditions by one-way ANOVA and a Tukey post hoc test. **D** Western blot shows Dox-induced expression of the indicated constructs and αFlag IPs in THP1 AML cells. **E** Representative flow cytometry plots. **F** Heatmaps show ChIP signal for the indicated proteins and conditions. Line graphs above show mean normalized ChIP signal for each heatmap surrounding all (black line) or the strongest 20% (red line) of HMG20B peaks. **G** Exemplar ChIPseq peaks tracks.

Assembly of the GFI1:LSD1 transcription repressor complex on chromatin is, like other complexes, likely to be dynamic with variable chromatin scanning and residence times of individual proteins determining stability of the whole. We hypothesized that by directly linking HMG20B to the DNA-binding domain of GFI1 (GFI1-ZNF) the resulting GFI1-ZNF-HMG20B fusion protein would serve as a stable platform for recruitment of LSD1 by circumventing a requirement for co-localization of separate full-length HMG20B and GFI1 proteins at the same site. Making use of conditional constructs (Fig. 4A), immunoprecipitation experiments confirmed that as expected GFI1.ZNF-HMG20B interacted with both LSD1 and RCOR1 (Fig. 4B).

We previously demonstrated that LSD1 inhibitor-induced upregulation of a myeloid differentiation program in THP1 AML cells is dependent upon drug-induced physical separation of LSD1 from GFI1 rather than inhibition of the enzymatic activity of LSD1, and that expression of a GFI1.ZNF-LSD1 fusion protein which is resistant to drug-induced physical separation is sufficient to confer cellular resistance to LSD1 inhibitors [8]. Our experiments here show that AML cells exposed to OG86 250 nM or DMSO vehicle for 48 h undergo the expected upregulation of myeloid differentiation markers CD11b and CD86, and that this is unaffected by induced expression of either the DNA-binding domain of GFI1 (GFI1-ZNF) or HMG20B alone. In contrast, induced expression of the GFI1.ZNF-HMG20B fusion protein completely blocked CD11b and CD86 upregulation (Fig. 4C–E). To confirm this was due to stabilization of LSD1 on chromatin by the induced fusion protein, we performed ChIPseq. While ChIP signal for Flag-HMG20B and LSD1 decreased following exposure of cells to OG86 250 nM, in the presence of the GFI1-ZNF-HMG20B fusion LSD1 ChIP signal was largely retained (Fig. 4F, G). The interaction of LSD1 with the GFI1.ZNF-HMG20B fusion protein, and thus its recruitment to chromatin, was though the HMG20B coiled-coil domain (ΔCC) because the ΔCC GFI1.ZNF-HMG20B fusion protein mutant failed to interact and was unable to block the upregulation of CD11b or CD86 (Fig. 4C, D).

Thus, even in the absence of the SNAG domain sequence of GFI1, a fusion of the DNA-binding domain of GFI1 with HMG20B was sufficient to stably recruit LSD1 to chromatin in the presence of an LSD1 inhibitor to maintain transcription repression.

### HMG20B stabilizes LSD1:GFI1 interaction on chromatin preventing enhancer activation

**Fig. 5:**
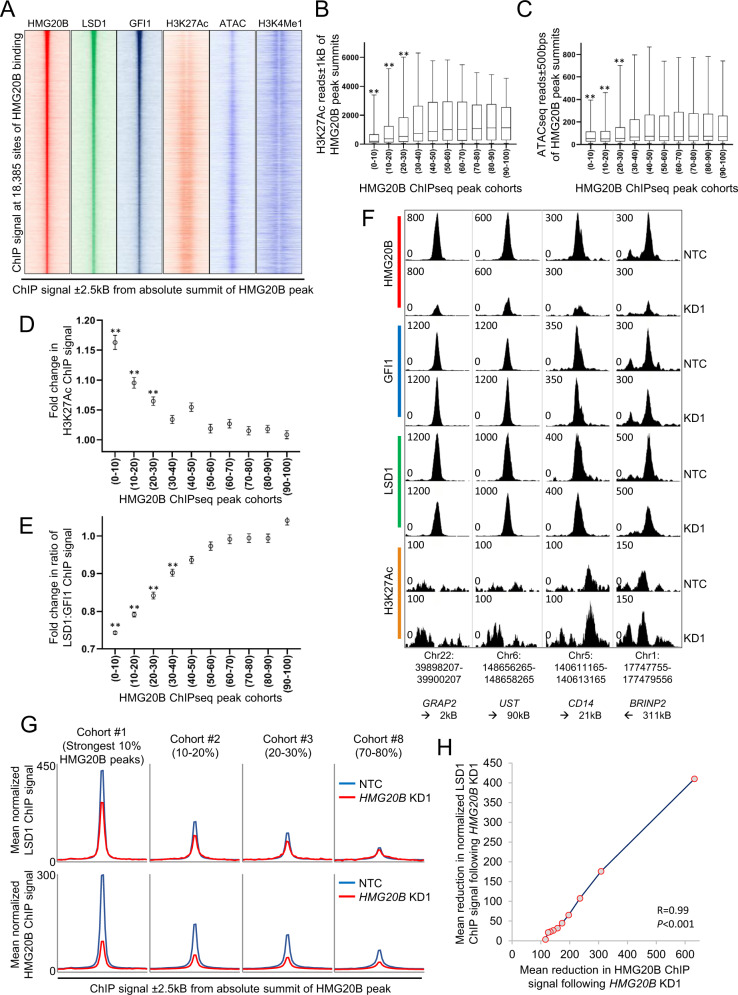
HGM20B stabilizes the interaction of LSD1 with GFI1 on chromatin. **A** Heatmaps show ChIP signal for the indicated proteins, histone modifications, and chromatin accessibility. **B**, **C** HMG20B ChIPseq peaks were grouped into ten cohorts according to peak strength. Boxplots show median, 25th and 75th centile values (box), and 5th and 95th centile values (whiskers) for **B** H3K27Ac ChIP signal or **C** ATACseq signal surrounding the indicated cohort of HMG20B binding peaks. **D**–**F** THP1 AML cells were infected with lentiviral vectors targeting *HMG20B* for KD, or a non-targeting control (NTC). 72 h later ChIPseq was performed on puromycin-resistant cells. HMG20B ChIPseq peaks were grouped into ten cohorts according to peak strength. Graphs show mean ± SEM fold change in **D** H3K27ac ChIP signal or **E** normalized ratio of LSD1:GFI1 ChIP signal surrounding the indicated cohort of HMG20B binding peaks. For (**B**–**E**) ** indicates *P* < 0.01 for comparison of each of the top three cohorts versus each of the bottom five cohorts by one-way ANOVA and a Tukey post hoc test. **F** Exemplar ChIPseq tracks showing normalized ChIP signal. **G** Density plots show ChIP signal for (upper row) LSD1 (normalized to assume equal GFI1 ChIP signal in NTC and KD conditions) and (lower row) HMG20B in the indicated conditions and for the indicated exemplar cohorts. **H** Graph shows mean absolute loss of LSD1 ChIP signal versus mean loss of HMG20B ChIP signal (±300 bps from HMG20B peak summit) for the ten HMG20B cohorts after *HMG20B* KD.

Ahead of experiments to determine the functional importance of HMG20B to the LSD1:GFI1 interaction, we performed characterization of the chromatin and DNA accessibility surrounding HMG20B binding peaks. Consistent with the transcription repressor function of the GFI1/LSD1/HMG20B complex, among the strongest 30% of HMG20B ChIPseq peaks (corresponding to a threshold pileup value of ≥90), there was an inverse quantitative relationship between HMG20B peak strength and surrounding H3K27Ac ChIP and ATACseq signal (Fig. 5A–C). There was no relationship between HMG20B peak strength and surrounding H3K4Me1 ChIP signal (Fig. S3A). This suggests that chromatin binding of GFI1/LSD1/HMG20B below an HMG20B pileup value of ~90 had limited function, at least as far as direct or indirect repression of enhancer acetylation and chromatin accessibility is concerned.

To evaluate the consequences of HMG20B depletion upon the interaction between GFI1 and LSD1 on chromatin, as well as on surrounding histone acetylation, ChIPseq was performed for HMG20B, GFI1, LSD1 and H3K27Ac following in control and *HMG20B* KD cells. Fold change in normalized H3K27Ac ChIP signal surrounding each HMG20B peak was determined and then the mean fold change in H3K27Ac ChIP signal for each of the ten cohorts of HMG20B peaks ranked in order of peak strength (*n* = 1838 peaks per cohort). For the strongest three cohorts (corresponding to the strongest 30% of HMG20B ChIPseq peaks) there was a positive relationship between HMG20B peak strength and fold-change increase in the surrounding (±1kB from absolute peak summit) H3K27Ac ChIP signal following *HMG20B* KD (Fig. 5D). As a control for this analysis we calculated the fold change in H3K27Ac ChIP signal surrounding each of 24,479 binding peaks identified in THP1 AML cells for the transcription factor CEBPA following *HMG20B* KD (Fig. S3B, C). While there was a positive relationship between CEBPA peak strength and the level of surrounding histone acetylation, there was no relationship between CEBPA peak strength and fold change in H3K27Ac ChIP signal following *HMG20B* KD (Fig. S3B, C) demonstrating the specificity of the observed changes surrounding HMG20B binding peaks.

Next, to evaluate the consequences for GFI1 and LSD1 chromatin binding at HMG20B binding sites following HMG20B depletion, we performed a similar analysis and calculated the ratio of LSD1:GFI1 ChIP signal surrounding each HMG20B binding site (±300 bps from absolute peak summit) before and after *HMG20B* KD. Once more, above a threshold level of HMG20B peak strength (in this case the strongest four cohorts of HMG20B peaks, corresponding to the strongest 40% of HMG20B peaks (pileup value ≥78)) we observed a significant reduction in the mean ratio of LSD1 ChIP signal to GFI1 ChIP signal (Fig. 5E, F). This is further illustrated by the density plots shown in Fig. 5G. *HMG20B* KD led to localized reduction of LSD1 ChIP signal (normalized to assume constant GFI1 ChIP signal following KD), with the mean absolute reduction correlating strongly across HMG20B peak cohorts with the mean absolute reduction in HMG20B ChIP signal (Fig. 5H). Taken together, these data indicate that on a genome-wide basis HMG20B serves to stabilize the physical interaction on chromatin of LSD1 with GFI1.

### HMG20B is required for GFI1 repressor activity in normal and leukemic myeloid cells

**Fig. 6:**
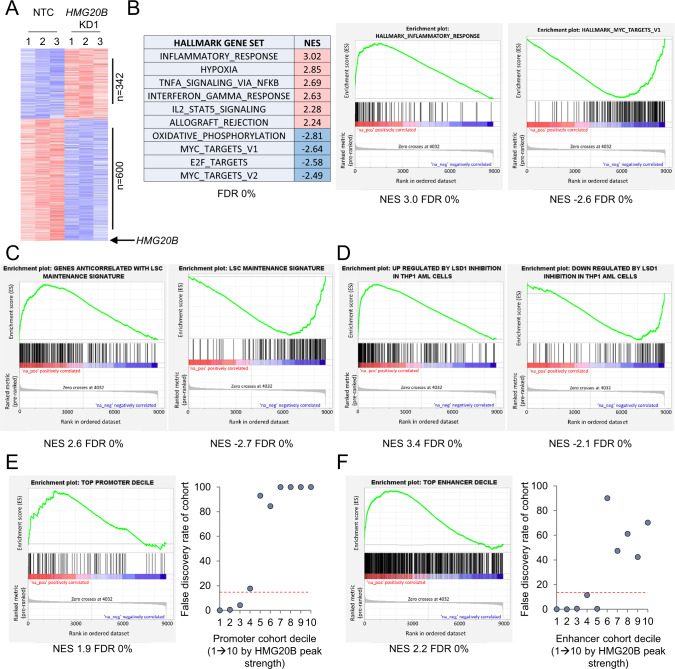
Gene expression changes induced by *HMG20B* knockdown. **A**–**F** THP1 AML cells were infected with lentiviral vectors targeting *HMG20B* for KD, or a non-targeting control (NTC) and selected for puromycin resistance. **A** Heatmap shows differentially expressed genes 72 h after initiation of KD. **B** Summary table and representative GSEA plots. **C**, **D** GSEA plots. **E**, **F** HMG20B binding peaks were grouped into ten cohorts according to peak strength and mapped to nearby genes to generate gene sets for use in GSEA. Graphs (right panels) show false discovery rates by cohort for genes bound by HMG20B at **E** promoters and **F** putative enhancers. GSEA plots are shown for the top cohorts (left panels).

To evaluate the consequences of *HMG20B* transcript depletion on the transcriptome, we performed *HMG20B* KD. Considering the 8733 expressed protein-coding genes (i.e., those with expression levels of 0.25 fragments per kilobase per million mapped reads (FPKM) in at least one of the six samples), 942 were differentially expressed 72 h after initiation of KD (Fig. 6A): 342 genes were upregulated and 600 downregulated (Fig. 6A and Table S5). We confirmed by qPCR that both *HMG20B* KD constructs induced increased expression of selected genes (*SPP1*, *BTG2, CD84* and *CD209*) identified as upregulated by RNAseq (Fig. S4). We made use of gene set enrichment analysis (GSEA) to confirm that *HMG20B* KD induced expression of a leukemia cell differentiation program. Using the Molecular Signatures Database Hallmark Gene Set collection (each of which conveys a specific biological state or process and displays coherent expression) [31] we noted that immune-associated gene sets which are highly expressed in terminally differentiated monocytes and macrophages (e.g., INFLAMMATORY_RESPONSE, INTERFERON_GAMMA_RESPONSE) were strongly enriched in *HMG20B* KD versus control cells. Likewise, those characteristic of cycling, metabolically active cells (e.g. E2F_TARGETS, MYC_TARGETS_V2) were depleted (Fig. 6B). In keeping with induction of a leukemia cell differentiation program we also noted that *HMG20B* KD resulted in strong depletion of a gene set which promotes leukemia stem cell maintenance in murine MLL-AF9 AML cells [32, 33]. There was also strong upregulation of genes whose expression is inversely correlated with leukemia stem cell activity (Fig. 6C). Most notably, comparison of the transcriptional changes induced by *HMG20B* KD with those observed following LSD1 inhibition in THP1 AML cells with OG86 [8] revealed a highly significant overlap (Fig. 6D). These transcriptional analyses provide further support to the concept that HMG20B is an essential cofactor involved in the repressive activity of GFI1, through stabilization of the interaction of GFI1 with LSD1 on chromatin.

To explore the link between HMG20B peak strength and change in gene expression following *HMG20B* KD, we mapped each of the 18,385 HMG20B binding peaks to the nearest gene using Genomic Regions Enrichment of Annotations Tool (GREAT) [34] and grouped them into 10 separate cohorts according to HMG20B peak strength. We considered peaks located at promoters or the 5′UTR of genes (“promoter peaks”), and those bound at intergenic or intronic regions (“enhancer peaks”), separately. Within the limitations of this approach – enhancers do not necessarily control expression of the nearest gene - we nevertheless observed that, among upregulated genes, the strongest enrichment was observed for genes mapping to the strongest decile of promoter-bound peaks and enhancer-bound peaks (Fig. 6E, F). In keeping with the observed changes in histone acetylation and the change in the GFI1:LSD1 ChIP signal ratio (Fig. 5D, E), there was also among upregulated genes significant enrichment of those mapping to HMG20B promoter peaks in the second and third decile (corresponding to a threshold pileup value of ≥ 90), and down to the fifth decile for the enhancer cohorts (down to a threshold pileup value of ≥ 70).

**Fig. 7:**
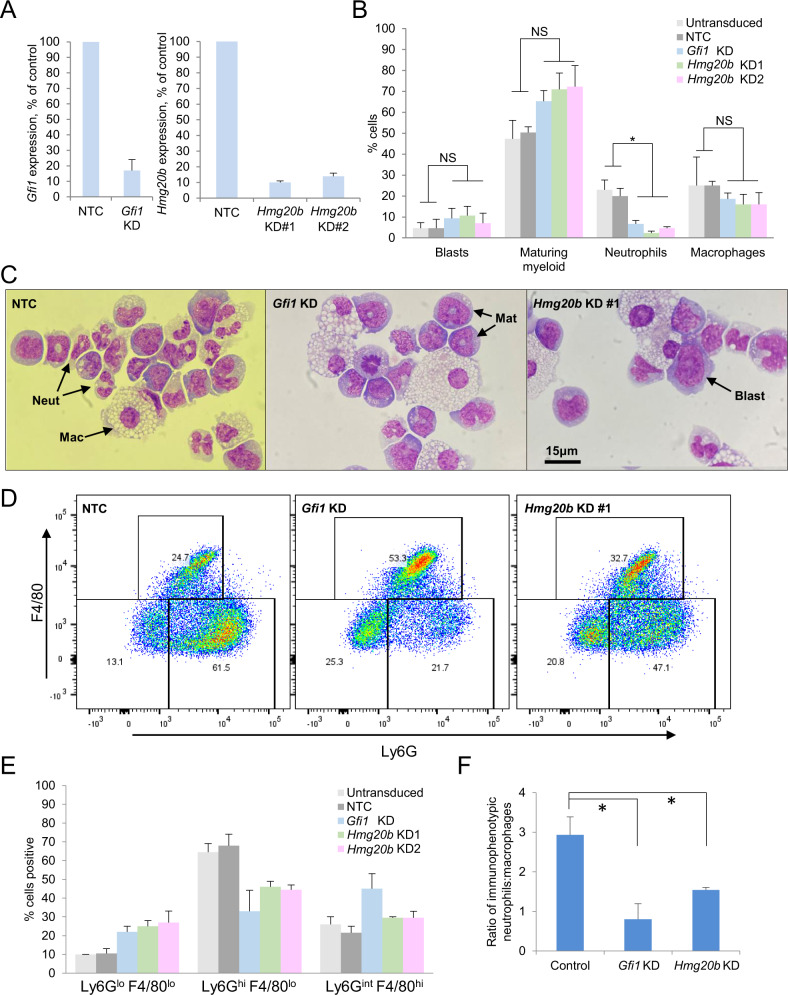
*HMG20B* knockdown in murine hematopoietic stem and progenitor cells. **A**–**C** Murine bone marrow KIT^+^ cells were infected with a lentiviral vector targeting *Hmg20b* or *Gfi1* for KD, or a non-targeting control (NTC), and selected with puromycin for 48 h prior to semisolid culture. **A** KD efficiency for the indicated genes at 72 h, as determined by qPCR. **B** Bar chart shows mean + SEM percentage of the indicated cell types in cytospin preparations after seven days in semisolid culture (*n* = 3). * indicates *P* < 0.05 for the indicated comparisons by one-way ANOVA with a Tukey post hoc test. **C** Exemplar cytospin preparations. Mat maturing, Mac macrophage, Neut neutrophil. **D** Exemplar flow cytometry plots. **E** Bar chart shows mean + SEM percentage of cells with the indicated immunophenotype after seven days in semisolid culture (*n* = 2); and (**F**) mean + SEM ratio of immunophenotypic granulocytes (Ly6G^hi^ F4/80^lo^) to macrophages (Ly6G^int^ F4/80^hi^). Control = untransduced or NTC (*n* = 4); *Hmg20b* KD = KD #1 and #2 (*n* = 4); *Gfi1* KD (*n* = 2). * indicates *P* < 0.05 for the indicated comparisons by one-way ANOVA with a Tukey post hoc test.

To provide additional evidence that HMG20B regulates myeloid lineage differentiation through stabilizing the interaction of LSD1 with GFI1 on chromatin, we performed KD experiments in murine KIT+ hematopoietic stem and progenitor cells (HSPCs) and cultured them in semisolid conditions favoring terminal differentiation. GFI1 is required for terminal granulocytic differentiation and so we hypothesized that *HMG20B* depletion would reduce the proportion of granulocytes in culture after seven days. As expected, control cells generated both granulocytes and macrophages while *Gfi1* KD substantially decreased granulocytic differentiation both by morphology and flow cytometry. Likewise, *Hmg20b* depletion also resulted in production of significantly fewer mature granulocytes (Fig. 7A–E). The ratio of immunophenotypic mature granulocytes to mature macrophages was also significantly lower following either *Gfi1* or *Hmg20b* depletion compared with control conditions (Fig. 7F). These observations are consistent with the concept that HMG20B serves a critical accessory role in the function of the transcription repressor GFI1.

## Discussion

Our studies reveal the coincident genome-wide binding of the HMG-box protein HMG20B with LSD1 and GFI1 in myeloid leukemia cells, and the functional relevance of the interaction of the coiled-coil domain of HMG20B with LSD1 in stabilizing the interaction of LSD1 with GFI1. Depletion of HMG20B was sufficient to trigger a leukemia cell differentiation program similar to that observed seen following treatment of leukemia cells with the LSD1 inhibitor OG86; and also to block granulocytic differentiation of normal murine hematopoietic stem and progenitor cells similar to that seen following *Gfi1* depletion.

While the location of the GFI1 transcription repressor complex is dictated by sequence-specific binding of the GFI1 zinc finger domain to its DNA consensus motif, the residency time of the assembled complex on chromatin, and thus the extent and duration of local transcription repression, is likely influenced by accrued co-location of additional co-factors. Our data suggest that HMG20B, which binds DNA independent of sequence via its HMG-box domain [30], may be one such factor. Other points of contact of the LSD1 complex with nucleosomal DNA include through the SANT2 domain of RCOR1 [35] and possibly through positively charged residues in the amino oxidase domain of LSD1 [36]. The role of other proteins identified as components of the LSD1 complex, such as ZMYM2, ZNF516, ZNF217, PHF21A, and RREB1, in stabilizing the whole and interacting with DNA remains largely unclear. It is also not clear whether there are important differences in function of LSD1 complex proteins according to whether the complex is tethered at repressed sites of SNAG domain transcription factor binding or, for example, at active OCT4-bound enhancers as is seen in embryonic stem cells [37].

Related to this, although recruitment of LSD1 is a prerequisite for GFI1-mediated transcription repression [20], a detailed understanding of how this results in transcription repression is lacking. It has been argued that recruitment of histone tail deacetylase, H3K4 demethylase, and H3K9 methyltransferase enzymatic activities by GFI1 may be sufficient to induce gene silencing [21]. However, (i) we identified neither EHMT1 or EHMT2 in our LSD1 or HMG20B pulldown experiments, (ii) the enzymatic activity of LSD1 is not required for transcription repression at sites of GFI1 binding [8], and (iii) expression of a GFI1 zinc finger-HDAC1 fusion protein only partially rescued GFI1-mediated transcription repression following treatment of AML cells with an LSD1 inhibitor [8]. It is possible that additional, as yet undetermined, mechanisms are of importance in GFI1-mediated transcription repression, such as phase separation and enhancement of RNA Pol II promoter pausing.

The role of HMG20B, and its paralog HMG20A, as components of the LSD1-repressor complex have been recently highlighted in other cellular contexts. In Merkel cell carcinoma, an aggressive neuroendocrine carcinoma of the skin, LSD1 inhibition derepresses key regulators of the neuronal lineage via the disruption of LSD1-CoREST complex integrity, including induction of HMG20B degradation [38]. Moreover, the dual depletion of HMG20B and HMG20A blocked GFI1B-mediated erythroid differentiation in the human erythroleukemia cell line K562 [27]. These data suggest that the involvement of HMG20B in stabilizing LSD1/CoREST may be a general mechanism. The human THP1 AML cell line used in our study showed little expression of the HMG20A paralog (data not shown), explaining why we did not identify HMG20A as an LSD1 co-partner. Interestingly, it has been proposed that HMG20A and HMG20B are mutually exclusive subunits of the complex [39], and in the developing central nervous system they display complementary expression patterns and functions [39]. Further studies are required to clarify the role of HMG20A in the CoREST complex in the hematopoietic system.

Our study adds to emerging data that further clarify the mechanisms of action of tranylcypromine-derivative LSD1 inhibitors such as iadademstat and bomedemstat which are currently being evaluated for efficacy alone or in combination in diverse malignancies such as AML, myelofibrosis and essential thrombocythemia [4, 40]. The potential of LSD1 inhibition to sensitize AML cells to other differentiation-inducing agents, like ATRA or BET protein inhibitors, has been postulated [3, 41, 42]. Interestingly, GFI1 may play a central role in some of these synergistic therapeutic effects. For instance, GFI1 expression is high in t(8;21) driven AML and is required for the growth of this AML subtype [43], which may explain the high sensitivity these cells show to LSD1 inhibition [44]. GFI1 has also been proposed to be functionally important in acute promyelocytic leukemia (APL). Its expression is directly regulated by PML/RARa and it coordinates with the fusion protein to maintain APL, in such a way that the PML/RARa-induced differentiation block can be relieved upon GFI1 knockdown [45]. Accordingly, LSD1 inhibition sensitizes AML cell lines to retinoic acid treatment through the blockage of LSD1:GFI1 interaction [46]. Finally, it is interesting to note that treatment of NB4 promyelocytic leukemia cells with LSD1 inhibitors leads to reduced association of the coiled-coil protein GSE1 with LSD1 [47, 48], as we also noted (Tables S1–3). This appears to be as a result of impaired protein translation, and GSE1 downregulation is required for LSD1 inhibitor-induced differentiation [48].

## Materials and methods

### Reagents and antibodies

Puromycin (#P8833), blasticidin (#15205) and doxycycline (#24390-14-5) were from Sigma (St Louis, MO). Trans-N-[(2-methoxypyridin-3-yl)methyl]-2-phenylcyclopropan-1-amine (OG86) was synthesized in house [2]. Details of other reagents, antibodies and biochemical methods are in the Supplementary Information.

### Human cells and cell cultures

The use of human tissue was in compliance with the ethical and legal framework of the UK’s Human Tissue Act, 2004. Primary human AML samples were from Manchester Cancer Research Centre’s Tissue Biobank (instituted with the approval of the South Manchester Research Ethics Committee; 18/NW/0092). Their use was authorized following ethical review by the Tissue Biobank’s scientific sub-committee and with the informed consent of the donor. THP1 cells were purchased from DMSZ (Braunschweig, Germany), verified by STR analysis and confirmed to be mycoplasma free. Details of cell culture methods are in the Supplementary Information.

### Expression constructs and lentiviral vectors

Details of vectors and cloning strategies are given in the Supplementary Information. Lentiviral supernatants were prepared, and human and murine cells were infected, as described previously [8].

### Mass spectrometry, ChIPseq, RNAseq, and data analysis

Mass spectrometry protocols, ChIPseq and RNA-seq protocols, and data analysis methods were as previously reported [8] and are as described in detail in the Supplementary Information.

## Supplementary information

Supplemental information

Table S5

## Data Availability

High throughput sequencing data files are available at Gene Expression Omnibus with accession number GSE192975. Mass spectrometry proteomics data are available via ProteomeXchange with identifier PXD030581.
