# Supplementary material for: HMG20B stabilizes association of LSD1 with GFI1 on chromatin to confer transcription repression and leukemia cell differentiation block
Source: Oncogene. 2022 Sep 28;41(44):4841–54. doi: 10.1038/s41388-022-02471-y (PMC7613766; doi:10.1038/s41388-022-02471-y)
Supplement: Supplementary file 1 — Supplemental information [file 41388_2022_2471_MOESM1_ESM.pdf]

Supplementary Information

HMG20B stabilizes association of LSD1 with GFI1 on chromatin to confer transcription repression and leukaemia cell differentiation block

Alba Maiques-Diaz, Luciano Nicosia, Naseer J Basma, Isabel Romero-Camarero, Francesco Camera, Gary J Spencer, Fabio MR Amaral, Fabrizio Simeoni, Bettina Wingelhofer, Andrew J.K. Williamson, Andrew Pierce, Anthony D Whetton and Tim CP Somervaille.

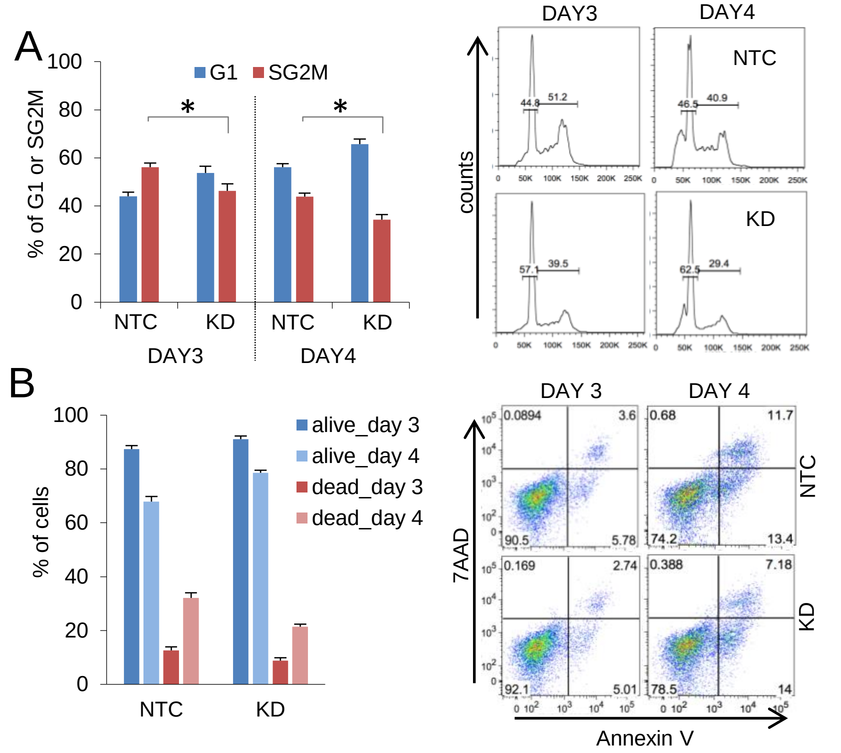

Figure S1. Depletion of *HMG20B* in primary patient *MLL* leukaemia cells.

Primary patient *MLL*-rearranged AML cells were infected with lentiviral vectors targeting *HMG20B* for knockdown (KD), or a non-targeting control (NTC), with puromycin drug resistance as the selectable marker. (A) Mean+SEM percentage of cells in G1 or SG_2M or (B) mean+SEM percentage of viable (alive) and dead cells (n=5 primary patient AML samples) in the indicated conditions and time points. Exemplar flow cytometry plots are shown to the right. Cells were cultured in stromal co-culture. * indicates *P*<0.05 by unpaired t-test. See also Figure 1.

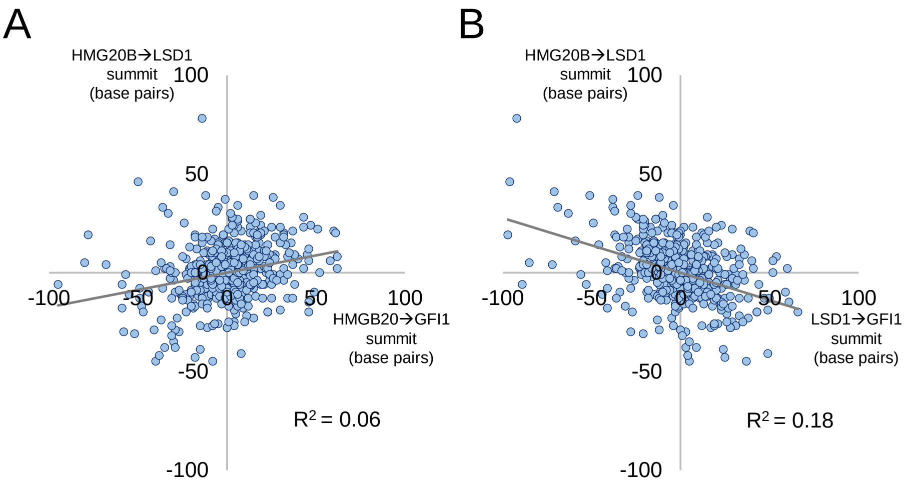

Figure S2. Colocalization of HMG20B with LSD1 on chromatin.

Dot plot show base pair distances between the absolute summits of HMG20B → LSD1 binding peaks versus base pair distances between the absolute summits of (A) HMG20B → GFI1 binding peaks, or (B) LSD1 → GFI1 binding peaks. See also Figure 3A.

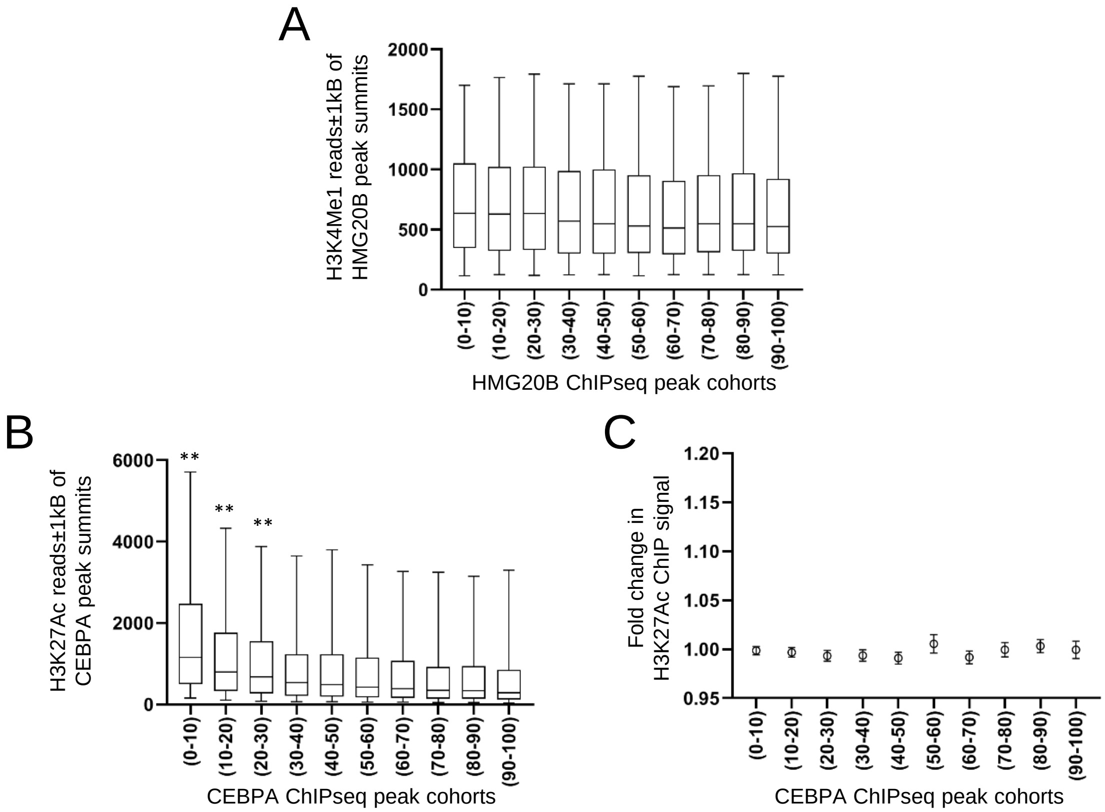

Figure S3. Chromatin surrounding HMG20B or CEBPA binding.

HMG20B or CEBPA ChIPseq peaks were grouped into ten cohorts according to peak strength. Boxplots show median, 25th and 75th centile values (box), and 5th and 95th centile values (whiskers) for (A) H3K4Me1 ChIP signal surrounding HMG20B peaks and (B) H3K27Ac ChIP signal surrounding CEBPA peaks for the indicated peak cohorts. (C) Graph shows mean±SEM fold change in H3K27ac ChIP signal surrounding the indicated cohort of CEBPA binding peaks. For (B) ** indicates P<0.01 for comparison of each of the top three cohorts versus each of the bottom five cohorts by one way ANOVA and Tukey post hoc test. No other comparisons were significant.

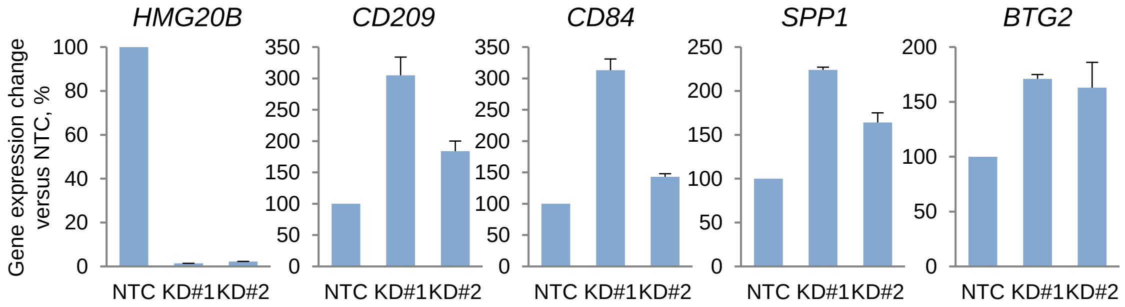

Figure S4. Validation of RNAseq expression changes for sample genes by qPCR.
Mean+SEM (triplicate analyses) of expression of the indicated genes following *HMG20B* KD with the indicated constructs in THP1 AML cells after 72 hours.

Supplementary Tables

Table S1: Proteins identified by LSD1 SILAC IP-MS from THP1 AML cells (peptides >10, excluding proteins likely to be contaminants - www.crapome.org). Proteins previously identified as part of an LSD1 complex are in bold red [1].

Uniprot ID	Gene ID	Gene Name	Peptides in LSD1 IP	Intensity DMSO	Intensity OG86	Relative intensity OG86:DMSO
**Q9UBW7**	***ZMYM2***	zinc finger MYM-type containing 2	40	31747000	20786000	1.2
**O60341**	***KDM1A***	**lysine demethylase 1A**	39	351800000	192530000	1
**Q92618**	***ZNF516***	zinc finger protein 516	38	24266000	16004000	1.21
**Q14687**	***GSE1***	Gse1 coiled-coil protein	35	84065000	26076000	0.57
**O75362**	***ZNF217***	zinc finger protein 217	26	15729000	11518000	1.34
**Q9UKL0**	***RCOR1***	REST corepressor 1	25	178410000	90902000	0.93
**Q15075**	*EEA1*	early endosome antigen 1	23	4132300	3290900	1.46
**Q92766**	***RREB1***	ras responsive element binding protein 1	22	15370000	4426000	0.53
**Q9P2K3**	***RCOR3***	REST corepressor 3	18	18581000	9121100	0.9
**Q13547**	***HDAC1***	histone deacetylase 1	18	82704000	43606000	0.96
**P04183**	*TK1*	thymidine kinase 1	16	175640000	83198000	0.87
**Q92769**	***HDAC2***	histone deacetylase 2	15	42524000	23500000	1
**Q9Y5B9**	*SUPT16H*	SPT16 homolog, facilitates chromatin remodeling subunit	15	3788100	2675900	1.29
**Q9P0W2**	***HMG20B***	high mobility group 20B	13	13691000	3929200	0.52
**Q96BD5**	***PHF21A***	PHD finger protein 21A	13	56192	24435	0.79
**P13674**	*P4HA1*	prolyl 4-hydroxylase subunit alpha 1	12	4113700	2142100	0.95
**F5H814**	***ZEB2***	zinc finger E-box binding homeobox 2	10	2050700	1527000	1.36

Table S2: Proteins identified by nuclear LSD1 IP-MS from THP1 AML cells (peptides >10 in at least one condition, excluding proteins likely to be contaminants - www.crapome.org). Proteins previously identified as part of an LSD1 complex are in bold red [1].

**Uniprot ID**	**Gene ID**	**Gene Name**	**DMSO # peptides**	**OG86 # peptides**	**IgG # peptides**	**Normalized relative ratio OG86:DMSO**
**O60341**	***KDM1A***	**lysine demethylase 1A**	79	80	0	1
**Q14687**	***GSE1***	Gse1 coiled-coil protein	54	25	0	0.46
**J3KN32**	***RCOR1***	REST corepressor 1	53	50	0	0.93
**Q92769**	***HDAC2***	histone deacetylase 2	43	36	2	0.84
**Q9P2K3**	***RCOR3***	REST corepressor 3	40	39	0	0.97
**Q92618**	***ZNF516***	zinc finger protein 516	39	42	0	1.06
**Q92766**	***RREB1***	ras responsive element binding protein 1	34	28	0	0.81
**Q14202**	***ZMYM3***	zinc finger MYM-type containing 3	33	28	0	0.84
**Q96BD5**	***PHF21A***	PHD finger protein 21A	32	30	0	0.93
**Q9UBW7**	***ZMYM2***	zinc finger MYM-type containing 2	30	34	0	1.12
**Q13547**	***HDAC1***	histone deacetylase 1	21	21	1	0.99
**O75362**	***ZNF217***	zinc finger protein 217	19	29	0	1.51
**Q9HAN9**	*NMNAT1*	nicotinamide nucleotide adenylyltransferase 1	17	21	0	1.22
**Q8IZ40**	***RCOR2***	REST corepressor 2	15	12	0	0.79
**P04183**	*TK1*	thymidine kinase 1	12	12	0	0.99
**Q8IX12**	*CCAR1*	cell division cycle and apoptosis regulator 1	11	18	0	1.62
**Q9P0W2**	***HMG20B***	high mobility group 20B	10	2	0	0.2

Table S3: Proteins identified by nuclear HMG20B IP-MS from THP1 AML cells (peptides >8 in at least one condition, excluding proteins likely to be contaminants - www.crapome.org). Proteins previously identified as part of an LSD1 complex are in bold red [1].

Uniprot ID	Gene ID	Gene Name	DMSO # peptides	OG86 # peptides	IgG # peptides	Normalized relative ratio OG86:DMSO
**Q9P0W2**	***HMG20B***	high mobility group 20B	32	37	0	1
**O60341**	***KDM1A***	lysine demethylase 1A	28	13	1	0.40
**J3KN32**	***RCOR1***	REST corepressor 1	13	6	0	0.40
**P18615**	*NELFE*	negative elongation factor complex member E	11	7	0	0.55
**Q14687**	***GSE1***	Genetic suppressor element 1	9	6	0	0.58
**Q9P2K3**	***RCOR3***	REST corepressor 3	9	3	0	0.29

Table S4. Karyotypes of primary patient AML samples.

Biobank number	BM or PB	Karyotype
**104**	BM	46,XX,t(6;9;11)(p21;p22;q23)[6]/45,idem,der(15)t(15;17)(p11.2;q11.2),-17[4]
**108**	BM	46,XX,t(6;11)(q27;q23)[10]
**148**	PB	46,XY,t(6;11)(q27;q23)[10]/48,idem,+der(6)t(6;11),+21[4]
**160**	PB	46,XX,t(9;11)(p22;q23),der(21;22)(q10;q10),+der(21;22)[10]
**419**	PB	46,XX,t(1;22)(p21;p11.2),ins(10;11)(p12;q23q1?4)[10] (MLL gene rearrangement confirmed by FISH)

BM – bone marrow. PB – blood.

Supplementary Methods

Reagents and antibodies

Antibodies used for western blotting were as follows: anti-LSD1 (2184; NEB, Hertfordshire, UK; and ab17721; Abcam, Cambridge, UK), anti-ACTB (MAB1501; Millipore, Watford, UK), anti-RCOR1 (07-455; Millipore), anti-GFI1 (sc-8558; Santa Cruz; and ab21061; Abcam), anti-HMG20B (14582-1-AP, Proteintech, Manchester, UK), anti-FLAG tag (F3165; Sigma), anti-histone H3 (3638; NEB) and anti-Vinculin (V9131; Sigma). All were used at a dilution of 1:1000 except for anti-GFI1 (1:200), HMG20B (1:200) and anti-ACTB (1:10,000). Antibodies used for immunoprecipitation experiments were as above or anti-HMG20B (ab72302; Abcam), anti-LSD1 (ab17721; Abcam), normal IgG Rabbit (12-307; Millipore), IgG Mouse (12-371; Millipore) and IgG Goat (NI02, Millipore).

Human cells and cell cultures

THP1 cells were cultured in RPMI 1640 with 10% fetal bovine serum, 2mM glutamine and 1% penicillin/streptomycin antibiotics or methylcellulose (H4320, Stem Cell Technologies, Vancouver, BC) without supplemental growth factors. For semisolid culture, starting culture density was 10^3/ml. Colonies were enumerated 10 days later. Cryopreserved leukemic blast cells collected from the bone marrow or blood of patients at presentation were thawed and co-cultured on MS5 stromal with alpha-MEM with 12.5% heat inactivated horse serum, 12.5% fetal bovine serum, 200nM glutamine, 57.2µM β-mercaptoethanol, 1µM hydrocortisone and 1% penicillin/streptomycin supplemented with 20ng/ml GCSF, 20ng/ml IL3 and 20ng/ml TPO (Peprotech, London, UK). The same growth factor combination was used for primary cell semisolid culture.

Viral particle manufacture

Lentiviral supernatants were prepared, and cells were infected with viral particles as previously described [2]. Briefly, 293FT cells were plated in 10cm dishes at a density of 4.5×10^6 cells per dish in 9ml DMEM with 10% FBS. Next day cells were transfected with 21ug polyethylenimine (PEI), 4ug of the lentiviral construct, 2ug of pCMVD8.91 and 1ug of pMDG.2 in 1ml serum-free DMEM. Follow 30 minutes of room temperature incubation to allow the formation of DNA-PEI complexes the mixture was added dropwise to 293FT cells. Next day the medium was replaced with 10ml fresh DMEM+10%FBS. All viral supernatants were collected after 24 and 48 hours and filtered through a 0.45um filter prior to use. Lentiviral supernatants were either used immediately or stored long-term at -80°C. For cell transduction 8ug/ul of polybrene was added to lentiviral supernatants and cells were centrifuged for 30 minutes at 900g and 37°C followed by an overnight incubation. Next day cells were transferred to normal media, and antibiotic selection was started 24 hours following spinoculation.

Isolation and culture of CD117+ murine cells

Bone marrow (BM) cells were harvested from the long bones of four- to eight-week-old mice by crushing them with a mortar and pestle. Next CD117+ BM stem and progenitor cells were recovered using immunomagnetic beads and an AutoMACS Pro Device (Milteny Biotec, Bergisch Gladbach, Germany). Cells were incubated overnight in RPMI with 20% fetal bovine serum (R20) with 20ng/ml SCF, 10ng/ml IL6, 10ng/ml GM-CSF and 10ng/ml IL3 (Peprotech, London, UK) to promote cell cycle entry. Next day cells were spinoculated with lentiviral supernatant in the presence of cytokines and 8ug/ul polybrene (Millipore) for 30 min at 900xg and 37ºC. Following spinoculation, cells were incubated overnight with R20 with growth factors to allow for expression of antibiotic resistance in transduced cells prior to the addition of puromycin (3ug/ml). Cells were either (i) harvested for RNA 48 hrs later to confirm knockdown efficiency or (ii) transferred to semisolid culture for clonogenic assays at a starting culture density of 10^3/ml. Colonies were enumerated 7 days later and cells were analyzed by cytospin staining and flow cytometry.

Generation of expression and knockdown constructs

To generate expression constructs for (i) full length human HMG20B (1-317aa) (NM_006339.2), (ii) HMG20B lacking the C-terminal (1-135aa, ΔC or (iii) HMG20B lacking the N-terminal part (136-317aa, ΔN), the human *HMG20B* gene was PCR amplified from THP1 cell cDNA using oligonucleotides which introduced coding sequences for a C-terminal GSG linker and Flag tag:

HMG20B full length (WT):

F: gaattcaccatggattacaaggatgacgacgataagatgtcccacggccccaagcagc

R: tctagattacaggtgctcgctggcgacctg

HMG20B 1-135aa (ΔC):

F: gaattcaccatggattacaaggatgacgacgataagatgtcccacggccccaagcagc

R: tctagataagcccgcagctccttcatgta

HMG20B 136-317aa (ΔN):

F: gaattcaccatggattacaaggatgacgacgataagtaccagcagtctgaagcct

R: tctagattacaggtgctcgctggcgacctg

The PCR product was sub-cloned into pGEM-T and after sequence verification cDNAs were excised using *Eco*RI and *Xba*I and sub-cloned into pLentiGS-minCMV-TET-blasticidin [2].

To generate a GFI1 ZNF HMG20B fusion construct, fragments coding for FLAG-GFI1 ZNF and full length HMG20B were amplified from existing plasmids [3] using the following primer sets:

HMG20B:

F: tctagaatgtcccacggccccaagcagc

R: catatgttacaggtgctcgctggcgacctg

Flag-GFI1 ZNF:

F: gaattcaccatggattacaaggatgacgacgataagtcctacaaatgcatcaaatgcagc

R: gctagctttgagtccatgctgagtctctc

Fragments were sub-cloned into pGEM-T Easy (Promega) and sequence verified to confirm orientation. To assemble the fusion, GFI1 ZNF was linearized using NheI and NdeI and HMG20B was cloned in using XbaI and NdeI. The assembled fusion product was excised from pGEM-T using EcoRI and NdeI and sub-cloned into the corresponding restriction sites of pLentiGS-minCMV-TET-puromycin [2]. Individual rounds of site directed mutagenesis of GFI1 ZNF HMG20B in pLentiGS-minCMV-TET-puromycin generated GFI1 ZNF HMG20B coiled-coil domain mutant. Oligonucleotides used for the mutagenesis PCR reactions were:

HMG20B ΔCC (Δ190-257)

F:ggcggtgagcgcttcagtgaagatgggaac

R:gttcccatcttcactgaagcgctcaccgcc

To generate lentiviral knock down constructs, the pLKO.1 puro TRC cloning vector (Addgene plasmid #10878) was digested with AgeI and EcoRI and ligated with HPLC purified oligonucleotides previously annealed by incubating at 98°C for 5 mins with slow cooling to room temperature. Oligonucleotide sequences were:

Human *HMG20B*:

KD#1:

F:ccggcatcgtccgcatcaaggaaatctcgagatttccttgatgcggacgatgtttttg

R:aattcaaaaacatcgtccgcatcaaggaaatctcgagatttccttgatgcggacgatg

KD#2:

F:ccggagcatccctttagctttcaatctcgagattgaaagctaaagggatgctttttg

R:aattcaaaaaagcatccctttagctttcaatctcgagattgaaagctaaagggatgct

Mouse *Hmg20b*

KD#1:

F:ccgggaaagaagattctcccaaatgctcgagcatttgggagaatcttctttcttttttg

R:aattcaaaaagaaagaagattctcccaaatgctcgagcatttgggagaatcttctttct

KD#2:

F:ccggaggtctgcactgagaagattcctcgaggaatcttctcagtgcagacctttttg

R:aattcaaaaaaggtctgcactgagaagattcctcgaggaatcttctcagtgcagacct

Mouse *Gfi1*

KD#1:

F: ccggcggctcctacaaatgcatcaactcgagttgatgcatttgtaggagccgtttttg

R: **aattcaaaaa**cggctcctacaaatgcatcaa**ctcgag**ttgatgcatttgtaggagccg

To generate FLAG RCOR1 pGEM-3Z, FLAG HMG20B pGEM-3Z and FLAG HMG20B dC pGEM-3Z Cds were excised from pTRE2 plasmids and subcloned into EcoRI/Xba I sites of pGEM-3Z (Promega).

Flow cytometry

Flow cytometry analyses were performed using either an LSR Model II BD FACSArray (BD Biosciences, Oxford, UK) or a Novocyte (Acea Biosciences, San Diego, CA, USA) flow cytometer. Antibodies used were anti-human CD11b-PE and anti-human CD86-PerCP-eFluor710

(eBioscience, Hatfield, UK). Apoptosis was assessed using a BD Pharmingen APC Annexin V kit (Oxford, UK), according to the manufacturer's instructions. Propidium iodide cell cycle analyses were performed as described [3]. Throughout the study, geometric mean cell fluorescence values are used.

GST pull-down assay

Human HMG20B was synthesized in vitro using the TnT® Quick Coupled Transcription/Translation System (Promega, L1170), according to the manufacturer's instructions. The T7 Luciferase Control DNA present in the kit was used as negative control for the reaction and the subsequent pull-down assay. The LSD1-GST protein was expressed into Rosetta BL21 (DE3) Competent Cells (Novagen®) in the presence of 1mM IPTG for 6 hours. The pull-down assay was performed using the Pierce™ GST Protein Interaction Pull-Down Kit (21516, Thermo Fisher Scientific), according to the manufacturer's instructions.

To generate pGEX-6P1-GST-LSD1, the full length LSD1 coding sequence was PCR amplified and subcloned into the EcoRI/SalI sites of pGEX-6P1-GST (Amersham) using the following primers:

F:atagaattcatgttatctgggaagaaggcggc

R:atagtcgacttacatgcttggggactgctgtg.

Chromatin immunoprecipitation and next generation sequencing

Cells were cross-linked at room temperature using 1% formaldehyde. After 10 minutes the reaction was stopped by incubation for 5 minutes with 0.125M glycine. Cell pellets were washed twice with cold PBS containing protease inhibitors (Complete EDTA-free tablets, Roche, Basel, Switzerland). 100 million cells were used for all ChIPs except for H3K27Ac where 50 million cells were used. As per [4], nuclear lysates were sonicated using a Bioruptor Plus (Diagenode) for 6 cycles at high, 30 sec ON, 30 sec OFF settings. Immunoprecipitation was performed overnight at 20rpm and 4°C, with 100µl magnetic beads (Dynabeads (Protein G), Invitrogen, Carlsbad, CA) per 10µg antibody per 100 million cells. ChIP-grade antibodies were used as follows: LSD1 (ab17721), GFI1 (ab21061), HMG20B (ab72302), Flag (F3165; Sigma) and anti-acetyl-H3K27 (ab4729; Abcam; 5ug per ChIP). After washing six times with RIPA buffer (50mM HEPES pH 7.6, 1mM EDTA, 0.7% Na deoxycholate, 1% NP-40, 0.5M LiCl), chromatin IP-bound fractions were extracted at 65°C for 30min with elution buffer (50mM TrisHCl pH8, 10mM EDTA, 1% SDS) vortexing frequently. RNAse A (1mg/ml) and proteinase K (20mg/ml) were used to eliminate RNA or protein from samples. Finally DNA was extracted using phenol:chloroform:isoamyl alcohol extraction and precipitated with ethanol (adding 2 volumes of ice-cold 100% ethanol, 2 ul glycogen (20µg/µl) and 200mM NaCl) for at least 1 hour at -80°C. Pellets were washed with 70% ethanol and eluted in 50ul 10mM TrisHCl pH8.0.

ChIP DNA samples were prepared for sequencing using the Microplex Library Preparation Kit (Diagenode) and 1ng ChIP DNA. Libraries were size selected with AMPure beads (Beckman Coulter) for 200-800 base pair size range and quantified by Q-PCR using a Kapa

Library Quantification Kit (Kapa Biosystems). ChIPseq data were generated using the NextSeq platform from Illumina with 2x75bp Mid Output.

Reads were aligned to the human genome (hg38) using BWA-MEM v0.7.15. Reads were further filtered using Bedtools v2.25.0 to keep only reads that mapped to standard chromosomes and to remove reads mapped to blacklisted regions defined by ENCODE (http://mitra.stanford.edu/kundaje). The number of uniquely mapped reads per sample was 50-100 million. MACS2 (Model-based Analysis of ChIPseq, version 2.1.0) software was used to call peaks [5]. DMSO or OG86 treated input samples were respectively used as reference. A cut off of 0.01 False Discovery Rate (FDR) was used as a threshold and five duplicates were allowed (*callpeak -t Chip.bam -f BAMPE -c input.bam --keep- dup 5 -g hs -B -q 0.01)*. For motif analysis a window of ±500bp around peak summits was analyzed using Centrimo [6] with default parameters. The genomic coordinates of peak apices were set at the centers of 100bp regions to create BED files using the package GenomicRanges (version1.30.1) (R/Bioconductor) [7] and then used for evaluating the intersection of peaks between different ChIPseq experiments with the BEDtools package (version 2.25.0) [8]. All sequencing data files are available at the Gene Expression Omnibus: GSE192975.

RNA sequencing and data analysis

Total RNA was extracted from puromycin selected THP1 AML using QIAshredder spin columns and an RNeasy Plus Micro Kit (Qiagen, Manchester, UK). RNA quality was checked using the Agilent Bioanalyzer. Indexed total RNA libraries were prepared with an input of 500ng of total RNA and 10 cycles of amplification using the TruSeq Stranded Total RNA LT Sample Preparation Kit – Set A (with Ribozero Gold). Library quality was checked using the Agilent Bioanalyzer. Libraries were quantified by qPCR using the KAPA Library Quantification Kit for Illumina. 1.8 pM pooled libraries were loaded onto the NextSeq 500 and 2x75bp sequencing was carried out using a NextSeq 500/550 High Output v2 kit. Reads were aligned to the human genome (GRCh38) and gene annotated with its corresponding GTF files (GENCODE GRCh38) using STAR version 2.4.2a with the settings: –outFilterMultimapNmax 20, –outFilterType BySJout,–alignSJoverhangMin 8, –quantMode GeneCounts [9]. DESeq2 was used to perform differential gene expression analysis and calculate FPKM (fragments per kilobase of transcript per million mapped reads) values for each gene, counting only reads that mapped to exonic regions [10]. All sequencing data files are available at the Gene Expression Omnibus: GSE192975

Gene set enrichment analysis

Pre-ranked gene set enrichment analysis was performed with GSEA v2.0.14 software from www.broadinstitute.org/gsea [11]. Expressed protein coding genes were rank ordered according to log2 fold change in expression (Table S5). Gene sets were from [32].

Protein extraction, western blotting and immunoprecipitation

For western blotting, cells were lysed in a high salt lysis buffer (45mM HEPES (pH 7.5), 400mM NaCl, 1mM EDTA, 10% glycerol, 0.5% Nonidet P40, 6.25mM NaF, 20mM β-glycerophosphate, 1mM DTT and 1x Protease Inhibitor Cocktail (Roche)) and equal amounts of protein were loaded and separated by SDS-PAGE. For subcellular fractionation experiments, lysates were prepared using a Subcellular Protein Fractionation Kit for Cells, according to manufacturer's instructions (Pierce, Rockford, IL). For co-immunoprecipitation of whole cell extracts, cells were lysed in TNN Buffer (50mM Tris-HCl (pH 7.5), 100mM NaCl, 5mM EDTA, 0.5% Nonidet P40, 6.25mM NaF, 20mM β-glycerophosphate, 1mM DTT, 1x Protease Inhibitor Cocktail (Roche), 10uM N-ethylmaleimide and 1:10,000 benzonase (Sigma) and lysate was incubated with the appropriate antibody pre-bound to Protein G–Sepharose (Sigma) or Dynabeads Protein G (10004D, Invitrogen) for two hours. Immune complexes were pulled down for two hours and washed four times with TNN buffer, eluted at 70ºC for 10 minutes or 95ºC for 5 minutes and separated by SDS-PAGE Western blotting. For co-immunoprecipitation of nuclear protein extracts the Nuclear Extraction Protocol (Invitrogen, Carlsbad, CA) was used. Briefly, cells were lysed in hypotonic buffer (20mM Tris-HCL, Ph7.4, 10mM NaCl, 3mM $MgCl_2$ containing 1x Protease Inhibitor Cocktail (Roche)) and 10% NP40 was added followed by centrifugation for 10 min at 3000rpm at 4ºC. The nuclear fraction pellet was resuspended in cell extraction buffer (Invitrogen) (with 6.25mM NaF, 20mM β-glycerophosphate, 1mM DTT, 1x Protease Inhibitor Cocktail (Roche) and 1:10.000 Benzonase) for 30min on ice followed by centrifugation for 30min at 14,000 x g at 4ºC. The nuclear fraction supernatant was used for immunoprecipitation, as described above. Horseradish peroxidase-linked secondary antibodies (GE Healthcare, Little Chalfont, UK) and ECL (GE Healthcare) or SuperSignal (Pierce) were used to detect immune complexes. Protein expression levels were quantified using Image J software v1.74c (NIH, USA).

Cytospin analyses

2-5x10^4 cells were resuspended in 150µl PBS and, through centrifugation at 60xg for 5 min, were spun onto a microscope glass slide and left to air dry. Cells were fixed by incubation in methanol for 10 minutes followed by May-Grunwald (Sigma; diluted 1:1 with Sorenson's Buffer (33.3mM KH_2PO_4, 64.75mM Na_2HPO_4, pH 6.8)) staining for 20 minutes and subsequent staining with Giemsa (Sigma; 10x diluted with Sorenson's Buffer) for 30 min. Finally, stained cells were washed under running tap water and left in Sorenson's buffer for five minutes prior to one final brief wash with tap water. Slides were left to air dry before cells were permanently mounted with a coverslip and DPX neutral mounting media (VWR, Radnor, PA). Images were obtained using a Leica SCN400 histology scanner (Leica, Solms, Germany) and analyzed using SlidePath Gateway software v1.0 (Leica).

Quantitative PCR

For quantitative PCR, first strand synthesis was performed using a High Capacity cDNA Reverse Transcription kit (Applied Biosystems, Paisley, UK). Quantitative PCR assays were performed in triplicate in a 384-well MicroAmp optical reaction plates using Taqman Fast Universal PCR Mastermix (Applied Biosystems) and the Universal Probe Library System (Roche) designed primers and probes; or SYBR Green, using 2x SYBR Green Mastermix. In all reactions done with SYBR Green the specificity of each couple of primers were assessed running a melting curve. Human and murine primers and probes are listed below (5'-3'):

- *HMG20B* Forward gagaaggggtcccacgag
- *HMG20B* Reverse gcagaatcttcttccgcttc probe 28
- *GFI1* Forward tctcggattgggtgtcaag
- *GFI1* Reverse ctgtcacccacggtcactc probe23
- *ACTB* Forward attggcaatgagcggttc
- *ACTB* Reverse ggatgccacaggactccat probe 11
- *Hmbg20b* Forward cggcttctgttccagatcc
- *Hmbg20b* Reverse gcttgacagccactacaaagg probe 71
- *Gfi1* Forward atgtgcggcaagaccttc
- *Gfi1* Reverse acagtcaaagctgcgttcct probe 1
- *Actb* Forward tgacaggatgcagaaggaga
- *Actb* Reverse cgctcaggaggagcaatg probe 106
- *CD209* Forward aagtaaccgcttcacctgga
- *CD209* Reverse cttcctccccaacgttgttg SYBR green
- *CD84* Forward tcagaaacagcacccgtagt
- *CD84* Reverse cttggtggtggtgtagggat SYBR green
- *SPP1* Forward cagtgatttgcttttgcctcc
- *SPP1* Reverse attctgcttctgagatgggtc SYBR green
- *BTG2* Forward gcagaggcttaaggtcttcag
- *BTG2* Reverse cttgtggttgatgcgaatgc SYBR green

ΔCt values relative to *ACTB* or *Actb* were assessed using SDS software v2.1 (Applied Biosystems).

SILAC and nuclear immunoprecipitation-mass spectrometry and analysis

The stable isotope labeling with amino acid in cell culture (SILAC) of THP1 cells was done as previously reported [12]. Briefly, cells were grown in heavy methionine and arginine or normal/light labeled media for 15 days (7 passages), and complete isotopic labeling was confirmed by mass spectrometry. Heavy-labeled cells were exposed to 250nM of OG86 and the light-labeled to DMSO control for 48 hours and 40 million cell per condition were used to extract total protein lysates with TNN buffer (50mM Tris-HCl (pH 7.5), 100mM NaCl, 5mM EDTA, 0.5% Nonidet P40, 6.25mM NaF, 20mM β-glycerophosphate, 1mM DTT, 1x Protease Inhibitor Cocktail

(Roche), 10uM N-ethylmaleimide and 1:10,000 benzonase (Sigma). The lysates from heavy-OG86 or light-DMSO were combined and incubated with the anti-LSD1 or anti-IgG rabbit antibodies pre-bound to Protein G–Sepharose (Sigma) for two hours. After washing four times with TNN buffer, proteins were eluted with Laemmli buffer, resolved using SDS-PAGE and each gel lane chopped into 10 pieces. Next, proteins were trypsinized and extracted from the gel for mass spectrometry analysis. Data were analyzed using Max Quant. The final list of interacting proteins was manually cleaned and we excluded any protein that had <10 peptides in the DMSO control, likely contaminants (based on the www.crapome.org list), or those with more than 2 peptides in the IgG control. We observed that the ratio of intensity between heavy-OG86 vs light-DMSO samples for KDM1A itself was 0.55, which suggest that less protein is pulled down in the presence of the inhibitor. Thus, we normalized everything to the KDM1A heavy/light ratio.

For nuclear immunoprecipitation followed by mass spectrometry (IP-MS) 80 million THP1 cells were cultured for 48 hours with 250nM OG86 or DMSO vehicle. Nuclear Extraction Protocol (Invitrogen, Carlsbad, CA) was used as described above. The nuclear lysates were then pulled down using with the anti-HMG20B (ab72302; Abcam), anti-LSD1 (ab17721; Abcam) or normal IgG Rabbit (12-307; Millipore) pre-bound to Protein G–Sepharose (Sigma) for two hours. Immune complexes were pulled down for two hours, washed four times with TNN buffer and subjected to on-bead digestion: 750µl trypsin reaction buffer was added with gentle agitation (300rpm) at room temperature for 5 minutes. Beads were spun down at 1700g and the supernatant was discarded. This step was repeated three times. 100µl trypsin reaction buffer with 500ng trypsin were added and the solution was left on the shaker at 37°C overnight to allow peptide fragmentation. The digest was aspirated away from beads, acidified with addition of TFA to 0.5% (v/v) and vacuum centrifuged to concentrate. Dried peptides were resuspended in 5ul 0.05% (v/v) TFA for liquid chromatography – mass spectrometry/mass spectrometry (LC-MS/MS). Each sample was eluted and run in triplicate, with peptides loaded directly in 1% acetonitrile 0.1% (v/v) formic acid using an RSLC nano HPLC system (Thermo). Peptides were separated and sprayed into the Orbitrap Fusion mass spectrometer (Thermo). The data was analyzed using Mascot (Matrix Science, London, UK; version 2.6.0). Mascot was set up to search the ens_homo_sapiens_86 database assuming the digestion enzyme trypsin. Mascot was searched with a fragment ion mass tolerance of 0.60 Da and a parent ion tolerance of 10.0 PPM. Scaffold (version Scaffold_4.8.4, Proteome Software Inc., Portland, OR) was used to validate MS/MS based peptide and protein identifications. Peptide identifications were accepted if they could be established at greater than 95.0% probability by the Scaffold Local FDR algorithm. Protein identifications were accepted if they could be established at greater than 99.0% probability and contained at least 2 identified peptides. Protein probabilities were assigned by the Protein Prophet algorithm [13]. Proteins that contained similar peptides and could not be differentiated based on MS/MS analysis alone were grouped to satisfy the principles of parsimony. Proteins sharing significant peptide evidence were grouped into clusters. Total list of proteins were identified from the total unique peptides called, and the number of peptides obtained combining the three technical triplicates. The final list of interacting proteins was manually cleaned and we excluded any protein that was likely to be a

contaminant (based on the www.crapome.org list) or which showed more than 2 peptides in the IgG control. Moreover, we retained as true LSD1 interactors those proteins showing >=10 peptides in the DMSO control. For HMG20B we kept as true interactors proteins showing >=8 peptides in the DMSO control.

Mass spectrometry proteomics data are available via ProteomeXchange with identifier PXD030581.

Statistics

Statistical analyses were performed using Microsoft Excel 2007 or StatsDirect software (v.1.9.7) (StatsDirect, Altrincham, UK). All data met the assumptions of the statistical tests used and variance was similar between groups.

Supplementary references

1. Lynch JT, Harris WJ, and Somervaille TC. LSD1 inhibition: a therapeutic strategy in cancer? Expert Opin. Ther. Targets. 2012; 16, 1239–1249.
2. Huang X, Spencer GJ, Lynch JT, Ciceri F, Somerville TD, and Somervaille TC. Enhancers of Polycomb EPC1 and EPC2 sustain the oncogenic potential of MLL leukemia stem cells. Leukemia. 2014; 28, 1081–1091.
3. Maiques-Diaz A, Spencer GJ, Lynch JT, Ciceri F, Williams EL, Amaral FMR, et al. Enhancer Activation by Pharmacologic Displacement of LSD1 from GFI1 Induces Differentiation in Acute Myeloid Leukemia. Cell Rep. 2018; 22(13):3641-3659.
4. Lee TI, Johnstone SE, Young RA. Chromatin immunoprecipitaion and microarray-based analysis of protein location. Nature Protocols. 2006;1, 729-748..
5. Zhang Y, Liu T, Meyer CA, Eckhoute J, Johnson DS, Bernstein BE, et al. Model-based analysis of ChIPseq (MACS). Genome Biology. 2008; 9, R137.
6. Bailey TL, Machanick P. Inferring direct DNA binding from ChIP-seq. Nucleic Acids Res. 2012; 40(17):e128.
7. Lawrence M, Huber W, Pages H, Aboyoun P, Carlson M, Gentelman R, et al. Software for Computing and Annotating Genomic Ranges. PLoSComputBiol. 2013; *9*: e1003118.
8. Quinlan AR, Hall IM. BEDTools: a flexible suite of utilities for comparing genomic features. Bioinformatics. 2010;26(6):841-2.
9. Dobin A, Davis CA, Schlesinger F, Drenkow J, Zaleski C, Jha S, Batut P, et al. STAR: ultrafast universal RNA-seq aligner. Bioinformatics. 2013;29(1):15-21.
10. Love MI, Huber W, Anders S. Moderated estimation of fold change and dispersion for RNA-seq data with DESeq2. Genome Biol. 2014;15(12):550.
11. Subramanian A, Tamayo P, Mootha VK, Mukherjee S, Ebert BL, Gillette MA, et al. Gene set enrichment analysis: a knowledge-based approach for interpreting genome-wide expression profiles. Proc Natl Acad Sci U S A. 2005; 102, 15545-15550.
12. Ong SE, Mittler G, Mann M. Identifying and quantifying in vivo methylation sites by heavy methyl SILAC. Nat Methods. 2004;1(2):119-26.
13. Nesvizhskii AI, Keller A, Kolker E, Aebersold R. A statistical model for identifying proteins by tandem mass spectrometry. Anal Chem. 2003;75(17):4646-58.
